# Comparative Efficacy of Virtual Reality–Assisted Cognitive Behavioral Therapy Versus Yoga-Based Interventions for Reducing Performance Anxiety in Students: Protocol for a Randomized Controlled Trial

**DOI:** 10.2196/66112

**Published:** 2025-06-30

**Authors:** Cristina Maria Tofan, Liviu-Adrian Măgurianu, Magdalena Axinte, Alexandra Maria Găină, Marcel Găină

**Affiliations:** 1 Gheorghe Zane Institute for Economic and Social Research, Romanian Academy - Iasi Branch Iasi Romania; 2 University of Medicine and Pharmacy „Grigore T. Popa” Iasi Romania; 3 Socola Institute of Psychiatry in Iasi Iasi Romania

**Keywords:** performance anxiety, yoga, virtual reality, cognitive behavioral therapy, CBT, randomized controlled trial

## Abstract

**Background:**

Performance anxiety represents a significant challenge for students, manifesting as a fear of failure and difficulties managing examination-related concerns and academic pressures. According to recent statistics, anxiety affects >4% of the global population, underscoring the need for effective management methods. Studies indicate that yoga and virtual reality (VR) training can alleviate symptoms of performance anxiety.

**Objective:**

This trial aims to compare VR-assisted cognitive behavioral therapy (CBT) with yoga interventions to find effective treatments.

**Methods:**

A single-blinded randomized controlled trial will be conducted with 60 participants (n=30, 50% per intervention group) recruited from university and preuniversity counseling centers. The trial will measure whether the interventions reduce performance anxiety in students. Stratified randomization will be used to ensure equal distribution of baseline anxiety levels and gender across both groups. The primary outcome is a reduction in anxiety, measured using the State-Trait Anxiety Inventory-Y1 and State-Trait Anxiety Inventory-Y2 subscales. Secondary outcomes include emotional regulation and quality of life. Data will be collected at baseline, after the intervention, and during follow-up assessments. Statistical analyses will include parametric tests (eg, repeated-measures ANOVA and *t* tests) to compare anxiety reduction, emotional regulation, and quality of life across groups. The intention-to-treat approach will be applied to minimize bias due to participant dropouts. Sensitivity analyses will assess the robustness of the findings.

**Results:**

This study is planned to start in September 2025 and end in June 2026. VR-assisted CBT is expected to reduce anxiety very quickly, whereas yoga is predicted to have long-term benefits.

**Conclusions:**

More generally, this research highlights the need for new, accessible forms of mental health support. VR-assisted CBT is an emerging digital mental health intervention, and it enables individuals to access and explore anxiety in virtual, safe environments. In contrast, yoga is a more conventional, all-encompassing discipline that enhances mental health through physiological and psychological processes that may have long-term effects.

**Trial Registration:**

ClinicalTrials.gov NCT06639841; https://clinicaltrials.gov/study/NCT06639841

**International Registered Report Identifier (IRRID):**

PRR1-10.2196/66112

## Introduction

### Background

Performance anxiety represents a significant challenge for many students, manifesting as an intense fear of failure and difficulties managing concerns associated with examinations and other academic activities. Recent international statistics [1,2] identify anxiety as one of the most prevalent issues globally, affecting >4% of the world population. In this context, alternative anxiety-reducing methods, such as yoga and virtual reality (VR) training, have gained increased attention both academically and practically. Recent research suggests that yoga practices and training using VR techniques can play a crucial role in alleviating performance anxiety symptoms.

As a mind-body practice, yoga offers a holistic approach that can help reduce anxiety symptoms by regulating the autonomic nervous system and reducing stress responses. By integrating poses (asanas), breathing techniques (pranayama), meditation, and deep relaxation, yoga can modulate brain activity and cortisol levels, thereby improving mental health. Regarding yoga-specific techniques, several experimental studies indicate a significant reduction in anxiety and an overall improvement in well-being through practices of meditation and breathing exercises [3-6]. These studies suggest that these techniques reduce anxiety through physiological and psychological mechanisms, including cortisol reduction and enhanced emotional regulation, and are effective in various contexts (eg, high-performing music students as well as students with lower academic performance). These findings are partially supported by a meta-analysis that highlights the positive effects of yoga on psychophysiological parameters associated with performance anxiety, such as heart rate and its variability, while also noting that further studies are needed to confirm their usefulness in reducing clinically diagnosed anxiety [7].

Specific research on evaluation anxiety in the literature mainly includes general studies, with only a few examining anxieties in various performance situations. Zoogman et al [8] reported significant outcomes from yoga interventions, drawing from controlled clinical research with diverse contexts, indicating the global effectiveness of yoga programs on anxiety symptoms. Another literature review underscores the efficacy of yoga programs for children and adolescents aged <18 years [9]. Although these studies have several methodological limitations, their findings indicate a general reduction in anxiety. In addition, Khalsa et al [4] showed that a 6-week program for music students significantly reduced music performance anxiety, with notable outcomes both at the end of the program and compared to a control group. Erdoğan Yüce and Muz [10] tested an intervention program for young people aged 18 to 25 years, where a yoga program including asanas (physical poses), pranayama (breathing exercises), and meditation contributed to improved physical and mental health. Conducted over 4 weeks with 60-minute sessions, the quasi-experimental study found a significant reduction in state anxiety but not trait anxiety. The program’s social quality of life (QoL) improved due to group interactions during the sessions, although the number of yoga sessions was limited and insufficient for significant trait-level changes.

Although the broader literature indicates some limitations in yoga research, Li and Goldsmith [11] identified specific issues, such as small sample sizes, control groups, and random group allocation. In addition, they highlighted the importance of intervention duration in anxiety and stress outcomes. For example, Rocha et al [12] found the best yoga intervention outcomes after 6 months, significantly reducing anxiety and other psychological health aspects. Participants were yoga naive, and the results were measured alongside a control group at the beginning of the intervention and after 6 months. Shohani et al [13] noted significant results after a substantial number of sessions (eg, 12 sessions) on various mental health aspects, including anxiety. In another study, the authors proposed a program for testing in 2021 [14] and synthesized the literature by collecting data from experienced yoga practitioners. They concluded that at least 10 yoga sessions significantly reduced anxiety symptoms and improved sleep, with participants reporting decreased anxiety severity and improved sleep quality after 10 sessions without significant adverse effects. The findings suggest that yoga can serve as an effective complementary treatment for anxiety; however, more extensive clinical studies are required to confirm its efficacy. Finally, Nanthakumar [15] synthesized the literature and analyzed randomized clinical research, finding that yoga positively impacted anxiety and depression symptoms across 8 studies involving interventions from 4 to 6 months.

Research indicates effectiveness regarding the impact of VR-assisted psychoemotional support, particularly when specific cognitive behavioral techniques are implemented [16]. In a recent dissertation [17], safe exposure to a virtual concert auditorium helped student artists reduce state anxiety. Similarly, Bissonnette et al [18] tested these techniques on music students with performance anxiety, demonstrating that one hour-long sessions across 3 weeks significantly reduced performance anxiety. Following short-term cognitive behavioral therapy (CBT) principles, this approach’s effectiveness highlights the potential for fewer sessions (eg, 4 sessions in a controlled experimental protocol) [19]. Finally, recent meta-analyses [20] indicate the superior efficacy of VR therapies compared to control groups, although they do not exceed the effectiveness of in vivo exposure.

Existing literature not only suggests that both intervention programs are effective but also reveals numerous research limitations. Hoge et al [21] compared yoga with cognitive behavioral and educational programs but not VR-assisted techniques, showing that yoga and CBT are more significant than educational methods. Although yoga shows promising results among students, compared to VR-assisted techniques, it is studied in general context and very few studies are specifically comparing them. For instance, Chauhan et al [22] demonstrated the effectiveness of a yoga program for medical students using these techniques to address anxiety and mental health aspects. Moreover, there is limited research testing intervention program efficacy for both state and trait anxiety. For instance, Somere et al [23] found that online yin yoga sessions for 2 hours per week significantly reduced state anxiety after each session, although the results of trait anxiety were less conclusive. O’Shea et al [24] noted that CBT is the standard psychological treatment for anxiety disorders. However, some individuals either do not access the treatment or do not benefit significantly from CBT alone. Finally, yoga, as a holistic mind-body practice, is seen as a potential CBT complement, providing benefits such as reduction in stress, anxiety, and depression through mindfulness, breathing, and movement techniques.

Previous research on VR-assisted CBT (VR-CBT) has predominantly focused on its efficacy in treating social anxiety, phobias, and generalized anxiety, often comparing it to passive relaxation or mindfulness-based interventions. However, these comparisons have largely assessed meditation, guided breathing, and progressive muscle relaxation without incorporating structured movement-based interventions such as yoga. This study addresses a critical gap by directly comparing the effects of VR-CBT with yoga, which extends beyond passive relaxation through the integration of physical postures (asanas), controlled breathing techniques (pranayama), and ethical self-discipline (Yama and Niyama). Unlike meditation alone, yoga actively engages both the body and mind, promoting physiological regulation of stress through autonomic nervous system modulation and improving emotional regulation via structured movement and breathwork. Previous studies have shown that VR-CBT provides rapid symptom relief through immersive exposure and cognitive restructuring, while meditation and relaxation techniques tend to cultivate mindfulness without the added physical benefits of yoga. By assessing yoga as a distinct intervention, this study aims to determine whether its multimodal approach leads to more sustainable long-term reductions in performance anxiety compared to the short-term but potentially more immediate effects of VR-CBT. The findings will offer new insights into the comparative efficacy of digital and traditional mind-body interventions for academic anxiety, contributing to the development of more tailored therapeutic strategies.

### Goal of This Research

This research aims to evaluate and compare the effectiveness of 2 psychological intervention modalities—VR-facilitated exposure techniques based on cognitive behavioral intervention and yoga techniques involving body posture, breathing, and meditation—in reducing performance anxiety symptoms. In addition, the study seeks to investigate whether VR techniques lead to a more significant reduction in state anxiety, while yoga techniques are more effective in reducing trait anxiety.

## Methods

### Research Design

#### Overview

This study is structured as a parallel group randomized controlled trial (RCT) comparing 2 distinct interventions. It uses a stratified randomization approach to account for variations in baseline anxiety levels and gender.

#### Blinding

Outcome assessors and data analysts will be blinded to group assignments to minimize potential biases.

### Participant Recruitment

Participants will be selected voluntarily. They will be invited to participate in the study through referrals from psychologists and psychiatrists working with individuals on waiting lists at counseling and mental health centers. These include psychological counseling centers at universities and preuniversity institutions. Eligible participants will be identified from the waiting lists of online platforms where they registered for counseling or psychotherapy related to emotional difficulties. Once identified, they will receive an invitation to participate in the research project. After expressing their intent to participate, each participant will receive an informed consent form detailing the study’s specifics. Participants will be formally included in the study upon signing the consent form. Finally, participants will be randomly assigned to one of the proposed psychoemotional support groups. This allocation will be conducted through random assignment, ensuring balanced groups in terms of financial level, type of education, and clinical level of anxiety.

### Eligibility Criteria

#### Inclusion Criteria

According to the Romanian Youth Law Number 350 of July 21, 2006, our research targets the youth age group, which includes adolescents and young adults aged 14 to 35 years. Given that there are specific recommendations regarding participants being aged ≥15 years for using VR software and hardware programs, our program will include adolescents and young adults aged 16 to 35 years. Another essential criterion for inclusion in the research is diagnosis, that is, participants diagnosed with disorders within the anxiety spectrum of mental disorders, particularly performance anxiety, who are capable of understanding and providing consent to participate in the study. Participants lacking full decision-making capacity (eg, those with severe psychotic disorders, schizophrenia, or any other disorder affecting judgment and those who have attempted suicide) are not eligible for participation.

To be eligible, participants must present a clinical or subclinical level of anxiety symptoms, confirmed by medical or psychological evaluation. According to the *Diagnostic and Statistical Manual of Mental Disorders, Fifth Edition* and *the International Classification of Diseases, 11th Revision*, test anxiety can be included within the following broader anxiety disorders: generalized anxiety disorder, social anxiety disorder (SAD; social phobia), or specific phobia. Symptoms include worry on most days, for ≥6 months, related to numerous events such as academic performance, examinations, etc. Anxiety and worry are accompanied by at least 3 of the following symptoms: restlessness, fatigue, difficulty concentrating, irritability, muscle tension, and sleep disturbances. SAD involves fears or anxieties about one or more social situations in which the person is exposed to possible scrutiny by others. For students, this may involve oral examinations or class presentations. Social situations almost always provoke fear or anxiety and are avoided or endured with intense fear or anxiety. Regarding specific phobia, if it centers solely on examinations, then, depending on its intensity and duration, it may constitute a specific phobia. Generally, cases are recorded where fear or anxiety is excessive or disproportionate to the situation, as there is no real threat from the situation itself.

We have also identified the following additional important inclusion criteria: (1) enrollment in an academic center or preuniversity educational institution and placement on the waiting list at psychological counseling centers within universities or preuniversity institutions, (2) the ability to understand all aspects of the study and the preliminary commitment to adhere to its interventions through signing informed consent, and (3) understanding of both Romanian and English.

#### Exclusion Criteria

The research team has identified the following key exclusion criteria that could negatively interfere with and influence the study’s results: (1) lack of consent from a legal representative for minors, undergoing medical treatment, diagnosis of epilepsy, auditory or visual impairments, mobility challenges, spinal disorders, nonclinical levels of anxiety; (2) psychiatric conditions that impair sensory or kinetic abilities required for the intervention procedures; (3) history of cybersickness, photosensitivity crises, or seizures induced by intermittent light stimulation (specific to the VR cohort); and (4) several medical, technological, and biosocial limitations related to exposure to VR techniques. These are detailed in the informed consent provided in Multimedia Appendix 1 for each participant assigned to the *VR* experimental condition. The most important aspects include neurological disorders, visual impairments, vestibular disorders, photosensitivity, cardiovascular issues, pregnancy, severe mental health disorders, technological and environmental limitations, cognitive disorders, impaired judgment, the lack of adherence to past medical or psychological treatments, and language barriers. These criteria are detailed in Multimedia Appendix 1 for each participant in both versions of the informed consent.

### Procedure

#### Overview

Participants will be invited to attend one of the psychoemotional support groups where they will benefit from either VR-facilitated exposure techniques based on cognitive behavioral intervention modalities or techniques based on the combination of postures (asana), breathing (pranayama), and meditation. Before allocation to the psychoemotional support conditions, each participant will be assessed using a personal data sheet to consider relevant medical, social, and demographic data and exclusion criteria.

This study will use a comprehensive set of preassessment measures to ensure stratified randomization before group allocation. First, measures of both state and trait anxiety are used to ensure participants are grouped appropriately according to their baseline anxiety levels before intervention allocation. Levels of clinical anxiety and emotional regulation mechanisms relevant to anxiety management as well as specific manifestations of performance anxiety will be assessed before the start of the intervention program. Randomization into groups will be done after the initial assessment and will consider an equitable distribution, and the equitable distribution will be considered according to anxiety levels. Given the impact of baseline anxiety levels on treatment effectiveness, a stratified randomization technique is used. This protocol outlines that participants are first stratified based on their anxiety scores and gender before being randomized into the 2 intervention groups. Stratification ensures that individuals with high-trait anxiety and low-trait anxiety are evenly distributed across both treatment groups. Similarly, gender will be controlled to ensure that results are not confounded by gender-related emotional regulation tendencies. This type of stratification minimizes variance and ensures comparable starting conditions for both groups. Finally, this process controls for preexisting differences, leading to stronger statistical effects.

The participants will then start the psychoemotional support program and complete an anxiety level assessment at the end of each intervention session. At the end of the program, participants will complete the anxiety questionnaire again, along with an instrument assessing their comfort, discomfort, and the quality of the specific intervention techniques. To evaluate manifestations of performance anxiety, correlations between clinical anxiety and academic anxiety will be checked to indicate the strength and significance of their relationship. Details about the implementation of the techniques are presented in Table 1.

**Table 1:** Overview of intervention components for virtual reality (VR)–assisted cognitive behavioral therapy (VR-CBT) and yoga-based interventions.

Intervention and phase		Session components	Duration
**VR-CBT**			
	Introduction and familiarization (sessions 1-2)	Psychoeducation, familiarization with VR equipment, and guided relaxation exercises	20-30 min per session
	Active exposure (sessions 3-8)	Exposure to anxiety-inducing VR scenarios (eg, virtual presentations), cognitive restructuring, and relaxation	20-30 min per session
	Evaluation and feedback (session 9)	Review of selected VR scenario, QoLI^a^, debriefing, and feedback	20-30 min
**Yoga-based intervention**			
	Yoga practices (sessions 1-10)	Body postures (asanas), breathing techniques (pranayama), meditation, and relaxation exercises	60-90 min per session
	Final assessment (after the intervention)	QoLI, and self-report feedback on intervention experience	60 min

^a^QoLI: Quality of Life Inventory.

#### Data Monitoring

A data safety monitoring board (DSMB) will monitor data collection and protocol compliance. These will include interim analyses, if necessary, with predetermined termination procedures if safety issues or important emerging reported adverse events (AEs) call for study termination earlier.

Given the low-risk nature of the interventions (VR-assisted CBT [VR-CBT] and yoga) and the absence of pharmacological components, a formal independent data monitoring committee is not deemed necessary for this study. Both interventions are noninvasive, well-established, and widely used in clinical and wellness settings, minimizing the likelihood of severe AEs requiring external oversight. Instead, a DSMB will be responsible for overseeing participant safety, protocol adherence, and interim data reviews. The DSMB will be composed of 3 independent experts, including a clinical psychologist specializing in anxiety disorders, a statistician, and a researcher with expertise in VR interventions, ensuring multidisciplinary oversight from our team. This board will meet at prespecified intervals (eg, the midpoint and end of the study) to evaluate participant retention, dropout rates, and any reported adverse effects, such as cybersickness from VR exposure or physical discomfort from yoga sessions.

This study uses a systematic approach to collecting, assessing, reporting, and managing AEs and unintended effects related to the interventions. Given the noninvasive nature of VR-CBT and yoga, AEs are expected to be minimal, but potential risks such as cybersickness (VR-CBT), physical discomfort or strain (yoga), or emotional distress (both interventions) will be monitored. Participants will be instructed to report any AEs immediately during or after intervention sessions either verbally to facilitators or via structured postsession feedback forms. Solicited AEs will be actively monitored through weekly check-ins, while spontaneous reports will be recorded in an AE log maintained by the research team. The severity of reported events will be classified as mild (transient discomfort), moderate (requires temporary intervention modification), or severe (necessitates withdrawal from the study). In the event of a significant or unexpected AEs, the study team will conduct a risk-benefit assessment, and if necessary, the DSMB will review the case. Participants experiencing persistent distress or injury will be referred to appropriate clinical support services. All AEs and responses will be documented, reviewed in regular safety meetings, and included in the final study report, ensuring transparency and participant safety.

#### Auditing Procedures and Independence

To ensure adherence to protocol, data integrity, and participant safety, this study will undergo periodic internal audits conducted by an independent ethics and compliance committee at the 2 supporting academic institutions. Audits will occur at three key time points: (1) after initial participant enrollment, (2) midway through data collection, and (3) at the study’s conclusion before final analysis. The auditing process will assess protocol compliance, adherence to ethical guidelines, proper documentation of informed consent, accurate data collection procedures, and AE reporting. Any discrepancies or deviations identified during the audit will be reported to the principal investigator (PI) and the institutional review board (IRB) for corrective action. While this audit process is not fully independent from the sponsoring institution, it remains separate from the direct investigative team, reducing potential conflicts of interest. If significant concerns arise, an external auditor may be consulted to review trial conduct. The final audit findings will be documented in the study report and shared with regulatory authorities if required, ensuring transparency and research integrity.

### VR Cognitive Behavioral Intervention and Theoretical Background

The psychoemotional support intervention, facilitated by VR exposure techniques (6-10 sessions), uses Guided Meditation VR software and stand-alone Oculus Quest 2 and HTC Vive Flow hardware for alleviating performance anxiety. These consist of approximately 6 to 10 sessions of 20 to 30 minutes each of psychoemotional support based on cognitive behavioral techniques and integrative psychotherapy (with extension to a maximum of 1 h, if necessary). They will be carried out over a period of 2 weeks, up to a maximum of 1 month if it is not possible to organize the sessions in optimal conditions. After 1 month, the participants are reported as having withdrawn (in case of a request from the participants) or dropped out of the psychoemotional support program. The initial sessions (1-2 sessions) are designed for psychological assessment, psychoeducation, and familiarization with VR exposure techniques. In the next 2 to 3 sessions, different VR scenarios are tested to find a comfortable virtual environment for each patient. We test if patients agree that they are present in that generated virtual environment in a manner comparable to exposure in a natural environment (eg, a walk by the beach, in a forest, or an underwater itinerary). At this stage, subsequent exposure techniques will be developed using a combination of cognitive restructuring and relaxation methods, aimed at familiarizing participants with the technology and ensuring a pleasant experience. The next 1 to 2 sessions will continue with exercises already chosen, based on cognitive restructuring techniques combined with relaxation techniques. The last session is designed to review one of the exercises, assessing the quality of the technique and offering suggestions for improvement by going through a set of questions (eg, QoL through the QoL questionnaire). Each session concludes with a brief clinical assessment of anxiety using the State-Trait Anxiety Inventory (STAI) tool. Specific manifestations of performance anxiety are assessed.

VR-CBT represents an innovative psychotherapeutic intervention method in mental health, addressing traditional challenges by integrating VR technology to facilitate exposure and cognitive restructuring in a controlled, immersive environment. Frequently used for treating anxiety, phobias, obsessive-compulsive disorder, and depression, VR-CBT offers patients a captivating alternative where they can safely experience anxiety-inducing scenarios in a gradual and secure manner. Advantages and specific characteristics of VR-CBT based on recent studies detailing its applicability, benefits, and risks in managing mental health disorders and improving mental well-being are highlighted [25,26]. In recent years, VR-CBT has been recognized as a leading-edge technology in treating mental health disorders and is considered a significant innovation for mental well-being. For instance, the World Health Organization has included mental health in its global strategies, underscoring the need to develop innovative and accessible therapies for treating anxiety, depression, and related disorders [27]. Amid global challenges, such as the COVID-19 pandemic, VR-CBT promises to support traditional therapies by reducing physical and emotional barriers associated with exposure sessions [28].

A distinct feature of VR-CBT is its precise control over exposure. Unlike real-world exposure, VR allows therapists to create and customize therapeutic scenarios in a safe, repeatable environment while monitoring the patient’s physiological responses, including blood pressure and heart rate [29]. For instance, participants with social phobias can gradually be exposed to simulated social interactions, thereby reducing real-world anxiety and enhancing coping skills [30]. VR exposure aims to induce emotional responses such as those experienced in real situations but within a context where patients feel they can control and manage stressful scenarios. This increased control encourages patient compliance with therapy and reduces dropout rates, which is essential in treating complex mental disorders such as phobias and generalized anxiety [31].

Besides improving therapy compliance and effectiveness, VR-CBT significantly alleviates anxiety symptoms. Studies show that repeated and monitored exposure in VR environments helps reduce stress and anxiety levels in real-life situations [32]. For example, in a program for young people with anxiety disorders, VR therapy contributed to decreased anxiety levels and improved emotional regulation skills. Another notable advantage is VR’s ability to track patient gaze and reactions, which is an essential aspect for diagnosing symptoms that are difficult to observe, such as suspiciousness and misinterpretation of others’ intentions in psychotic disorders [33].

The effectiveness of VR-CBT was evaluated in an RCT where patients were divided into 2 groups; one group underwent VR therapy, and the other received traditional relaxation and meditation techniques [20]. The study showed a significant decrease in anxiety symptoms in the VR group, particularly regarding reduced somatic reactions and stress-related symptoms. These results suggest that VR-CBT can be an effective complementary method to conventional approaches, thereby reducing the need for anxiolytics and providing patients with long-term self-regulation techniques [34]. Furthermore, VR-CBT offers substantial potential for global application, being an accessible and cost-effective treatment option compared to conventional therapy. VR platforms are becoming more accessible, enabling increased access for patients who cannot participate in in-person therapies due to geographical barriers or severe anxiety [35].

### Yoga Intervention and Theoretical Background

This psychoemotional assistance intervention is facilitated through breathing techniques, meditation, and body postures (yoga). The program is designed to reduce anxiety and improve emotional well-being through a holistic mind-body approach. This program is a psychoemotional support intervention that incorporates yoga-based techniques, including breathing exercises, meditation, and body postures. The program runs for 2 to 4 weeks, depending on the scheduling of the sessions, with participants attending 6 to 10 sessions lasting 60 to 90 minutes each. If participants do not complete the program within 1 month, they are considered to have withdrawn.

The sessions include the following:

- Body postures (asanas) that include a sequence of yoga postures such as Tadasana (mountain pose); Surya Namaskar (sun salutation); and various standing and seated poses such as the tree pose, warrior pose, and triangle pose, aimed at promoting physical alignment and relaxation.
- Breathing techniques (pranayama) that include exercises to control and expand the breath, such as Bhastrika (bellows breathing), Ujjayi (victorious breathing), and specific breath locks (Bandhas) to regulate energy flow.
- Meditation and relaxation that includes techniques such as Shavasana (relaxation pose) and chanting of the AUM mantra promote mental calmness and relaxation.

As described in Patanjali’s Yoga Sutras, Yama and Niyama represent 2 fundamental sets of ethical and moral rules within the yoga system. These principles, which form the foundation of any practitioner’s spiritual evolution, are intended for advanced yoga practitioners and beginners. Patanjali structured the yoga system into 8 progressive stages, known as ashtanga, which include Yama (rules for interacting with others) and Niyama (rules of inner discipline) as the first 2 steps of this system. Essentially, Yama and Niyama aim to guide the practitioner toward moral and spiritual balance, regulating both external and internal behavior. Following these principles is vital for reducing negative influences that may affect the practitioner, thus hindering spiritual growth and mental calm.

Yama, or the external moral rules, refers to the behaviors a yogi should adopt in relationships with others. These rules are ahimsa (nonviolence), satya (truthfulness), asteya (honesty), brahmacharya (sexual restraint), and aparigraha (nonpossessiveness). They form an ethical code governing practitioners’ thoughts, speech, and actions, with the purpose of preventing mental and emotional disturbances that can arise from unbalanced social interactions. For example, ahimsa, or nonviolence, refers to the absence of physical violence and nonviolence in thoughts and words. Practicing this rule leads to harmony in human relationships and, consequently, to mental calm.

Niyama, in contrast, governs the individual’s relationship with themselves. These rules are saucha (purity), santosha (contentment), tapas (discipline), svadhyaya (self-study and study of sacred writings), and ishvarapranidhana (devotion to divinity). Following these rules, the yogi cultivates inner harmony, thus reducing internal factors that block spiritual and mental progress. For instance, santosha, or contentment, encourages the practitioner to find peace and balance regardless of external circumstances, contributing to a significant reduction in stress and anxiety.

The practices described in Yama and Niyama are not merely abstract ethical codes but practical tools that directly influence emotion management and mental calmness. They offer a psychological discipline framework that facilitates internal and external conflicts, which ordinarily lead to emotional disturbances. In the Yoga Sutras, Patanjali argues that these rules are essential foundations for any progress in practice, allowing the establishment of a mental equilibrium that makes exploring higher stages of yoga possible, such as dhyana (deep meditation).

### Sample Size Determination

Considering that the research aims to test and compare 2 types of interventions (VR-facilitated exposure and yoga techniques), using a small sample population allows a detailed initial analysis and a rigorous evaluation of the mechanisms involved without requiring significant resources or large samples. In the exploratory phase, this type of study can identify important preliminary trends and effects, which can later be validated through larger studies. The study involves continuous monitoring of anxiety and other psychological and neurobiological parameters, which requires considerable resources for each participant. A small sample enables closer and more detailed tracking of each participant, ensuring higher-quality data collection and avoiding uncontrolled variables. The proposed interventions, such as yoga techniques and VR exposure, are complex and require specific preparation for each participant. Thus, a smaller population allows for more efficient adaptation of the intervention to individual needs and optimal program implementation. In addition, studies of this type often require costly equipment and expertise, such as VR hardware and certified yoga instructors. Therefore, limiting the sample population allows for efficient use of available resources while maintaining intervention quality. Finally, it is worth noting that previous studies have shown that both yoga and VR interventions can yield significant results with a limited number of sessions and participants. This suggests that a small sample may be sufficient to detect the desired psychological effects in the initial phase. Considering power analysis, 30 participants per group are required to detect significant differences regarding anxiety reduction with 80% power at a 5% significance level.

In psychological intervention research, particularly in RCTs involving novel therapeutic modalities such as VR-CBT and yoga, smaller sample sizes can be justified based on several methodological and practical considerations. First, exploratory trials with focused intervention designs often require fewer participants to detect meaningful preliminary effects, as observed in previous studies on VR-assisted exposure therapy and yoga-based interventions for anxiety [20]. In addition, studies incorporating intensive within-subject monitoring, such as repeated anxiety assessments over multiple sessions, benefit from smaller samples to enhance feasibility and ensure high-quality data collection without excessive resource expenditure [35]. Research also indicates that both VR-based cognitive interventions and yoga can yield significant results with as few as 20 to 30 participants per group, particularly when using highly targeted measures such as the STAI-Y1 or STAI-Y2, physiological markers, and emotion regulation scales [25,32]. Moreover, the personalized nature of interventions such as VR-CBT and structured yoga programs allows for greater within-group effect detection, reducing the need for large-scale samples compared to broad-spectrum behavioral health studies [31]. Given these considerations, the chosen sample size of 30 participants per intervention group aligns with previous RCTs demonstrating efficacy in VR and yoga-based anxiety interventions while maintaining statistical power at an optimal level for detecting significant differences in anxiety reduction [36]. This methodological approach ensures that while the study remains manageable in scale, it retains the rigor necessary to provide valuable insights into the comparative effectiveness of digital and holistic anxiety-reduction strategies.

### Screening

The enrollment form is designed to enable the selection of participants who meet the inclusion criteria and exclude those who do not meet the established research conditions. The introductory section of the form provides an overview of the research project and details the proposed program, including its duration, number of sessions, information about the research team and affiliated institutions, potential benefits in reducing anxiety, confirmation that the program is free, and clarification that there are no financial rewards. It also specifies the program’s purpose and how the research results will be used. Initially, we ensure that all respondents have read, understood, and agree with all the information provided in the introduction. The personal data section is followed by specific questions to determine eligibility for inclusion in or exclusion from the practice group. The first set of questions is designed to check alignment with the target age range and educational level (educational level, ongoing studies, the type of education, age, and financial level). The second set of questions assesses the mental and physical health of the participants and whether they are undergoing medication treatment or psychotherapy sessions, which are all essential considerations for inclusion in or exclusion from the research program. On the basis of the responses, those who meet the inclusion criteria will receive the initial evaluation test set.

### Instruments

#### Overview

First of all, participants will complete personal, medical, social, and demographic data sheets to identify aspects that make the volunteer incompatible with participation (ie, country of residence, county or region, gender or sex, level of education, educational attainment, current education, financial situation, marital status, profession, seniority in profession, level of responsibility in the workplace, and physical and psychiatric medical data, eg, current psychiatric diagnosis, undergoing medication, diagnosis of epilepsy, hearing or visual impairment, mobility difficulties, and spinal disorders).

#### Clinical Anxiety

Spielberger STAY-Y1 and STAY-Y2 subscales are used for the assessment of anxiety symptoms (the versions accredited by the Romanian College of Psychologists), which allow the identification of clinical symptoms of anxiety. The STAI (STAI-Y1 and STAI-Y2) [37,38] is a widely used instrument designed to measure both temporary anxiety (state) and general anxiety disposition (trait). The STAI-Y1 assesses state anxiety (situational emotional response), while the STAI-Y2 measures trait anxiety (general and enduring anxiety tendencies). The STAI has demonstrated high internal consistency (Cronbach α >0.90 for both subscales). It has been validated across multiple populations, including university students and patients with clinical anxiety. Extensive research supports its use in measuring performance-related anxiety and emotional dysregulation.

#### Performance Anxiety

The Academic Anxiety Inventory [39] is used for the assessment of performance anxiety manifestation, reflecting general patterns of anxiety related to examination situations or those related to solving specific academic tasks. Internal consistency is reported above 0.80, and construct validity has been demonstrated in studies linking cognitive interference, test anxiety, and academic performance [39].

#### Emotion Regulation Strategies

The Emotion Regulation Questionnaire [40] is a 10-item self-report scale measuring respondents’ tendency to regulate their emotions in two ways: (1) cognitive reinterpretation and (2) suppressing expression. We used a 7-point Likert-type scale ranging from 1 (strongly disagree) to 7 (strongly agree). Previous studies indicate good internal consistency, correlating well with measures of psychological well-being [41].

#### QoL Assessment

Tools will be used to assess the quality of the techniques used by assessing comfort or discomfort and the quality of psychoemotional support techniques. The QoL scale, through the QoL Inventory questionnaire, will be used.

### Data Analysis and Handling Missing Data

The analysis involves the use of advanced data management and statistical analysis software (eg, SPSS Statistics [IBM Corp] and Amos [IBM Corp]), and comparisons will be made between intervention groups using parametric statistical tests (eg, *t* tests and longitudinal or comparative analyses with repeated measures to determine participants’ progression). Initially, descriptive analyses of participants’ characteristics will be conducted, and the associations among research variables will be statistically analyzed (eg, Pearson correlations, partial correlations tailored to preliminary analyses, and group differences). We will use a method of controlling for additional variables that may influence statistical outcomes (eg, personal, medical, social, and demographic data) based on the results of the preliminary analyses.

The primary analysis (eg, participant randomization) will be based on the intention-to-treat principle. Missing data will be imputed using various techniques, and sensitivity calculations will compare the results to per-protocol analyses to determine the effect of dropouts.

### Ethical Considerations

This study has been approved by the University of Medicine and Pharmacy IRB Research Ethics Committee (466/2024-15.10) and will be conducted in full compliance with the Declaration of Helsinki, the World Medical Association’s ethical guidelines for human research, and relevant national and institutional research policies. These ethical safeguards ensure that participants’ rights, safety, and well-being are prioritized throughout the study.

Before enrollment, all participants will provide written informed consent, ensuring that they fully understand the study’s purpose, procedures, potential risks, and benefits. For participants aged <18 years, parental or legal guardian consent will also be required. The consent process will emphasize that participation is entirely voluntary, without compensation and individuals have the right to withdraw at any time without penalty or consequence. To maintain participant confidentiality, all collected data will be deidentified and coded, ensuring anonymity throughout the study. Data will be stored in secure, password-protected electronic databases, accessible only to authorized research personnel. Any publications or data-sharing initiatives will strictly follow ethical guidelines to prevent participant identification.

While both VR-CBT and yoga interventions are considered low risk, potential adverse effects will be monitored throughout the study. Participants will be encouraged to report any AEs immediately, and a clinical psychologist will be available to provide support as needed. Should a participant experience persistent distress or injury, they will be referred to appropriate medical or mental health services for further care. A structured protocol will be implemented for identifying, documenting, and managing AEs. Given the noninvasive nature of the interventions, a formal independent data monitoring committee is not required. Instead, a DSMB will be responsible for ensuring participant safety, protocol adherence, and data integrity. The DSMB will convene at predefined intervals (midstudy and at trial completion) to review participant retention, adherence, and safety outcomes. Findings will be documented and reported as needed. Any protocol deviations or concerns will be escalated to the PI and the IRB, with corrective actions implemented as necessary.

### Dissemination Policy

To ensure transparency and broad dissemination of trial findings, the study results will be communicated through multiple channels to participants, health care professionals, researchers, and the public. Upon study completion, participants will receive a summary of findings in lay language, detailing overall outcomes without compromising confidentiality. Results will also be submitted for publication in peer-reviewed journals and presented at academic conferences, mental health symposiums, and relevant professional meetings. In addition, findings will be reported in public clinical trial registries, such as ClinicalTrials.gov, in accordance with international reporting standards. The research team will explore open-access publication options to maximize accessibility. Where feasible, deidentified data may be shared upon request with other researchers for secondary analysis, following institutional guidelines on ethical data sharing. Any publication restrictions will be governed by funding agreements, journal policies, and ethical considerations, but the investigators and sponsors affirm their commitment to full, unbiased reporting of results, regardless of outcome.

Authorship for all study-related publications will follow the guidelines set forth by the International Committee of Medical Journal Editors, ensuring that all listed authors meet the criteria for substantial contributions to the study. Specifically, authorship will be granted to individuals who have (1) significantly contributed to the study design, data collection, analysis, or interpretation; (2) participated in drafting or revising the manuscript critically for intellectual content; and (3) approved the final version for publication. Individuals who contribute to the study but do not meet all authorship criteria, such as those providing administrative support, technical assistance, or general supervision, will be acknowledged in the acknowledgments section rather than listed as authors. The use of professional medical writers is not planned for this trial.

To promote transparency and reproducibility, the study team intends to provide public access to key study materials, including the full trial protocol, anonymized participant-level dataset, and statistical analysis code, where feasible and ethically appropriate. The final protocol will be made available through an open-access repository or as a supplementary file in peer-reviewed publications. Requests will be reviewed by the study’s ethics board and PI, ensuring that data align with ethical guidelines and data protection laws. In addition, the statistical code used for data analysis will be deposited in a publicly accessible repository (eg, GitHub and Open Science Framework) to facilitate replication and secondary analyses. Restrictions may apply if the data contain sensitive information that cannot be fully anonymized. Further details on data access and sharing policies will be outlined in the final study report and registered in public trial databases, such as ClinicalTrials.gov.

## Results

This study hypothesizes that the implementation of both VR techniques and yoga practices will yield a significant reduction in anxiety symptoms, as measured by the STAI-Y1 and STAI-Y2 scales. This study will start in the middle of the academic year in the fall of 2025 (to be relevant for performance anxiety) and will end in June 2026. It is anticipated that the VR group will experience a decrease in performance anxiety symptoms compared to the yoga group, which may require additional sessions. Concurrently, yoga sessions are expected to exert an intense impact with substantial psychoemotional rehabilitation effects. Furthermore, both interventions are predicted to lead to a meaningful enhancement in emotional regulation abilities, as assessed by the Emotion Regulation Questionnaire, with participants engaged in yoga anticipated to develop more effective cognitive reappraisal strategies. In assessing QoL, the use of the QoL Inventory among participants aims to reveal improvements, particularly in areas related to academic stress and examination-related anxiety, with both groups likely to report enhanced QoL after the intervention.

Comparative analyses between the 2 groups will highlight the specific outcomes associated with each technique. It is expected that the VR intervention may offer a more significant advantage in reducing acute anxiety symptoms, with rapid effects on stress perception. At the same time, the yoga group may sustain these effects over the long term by learning self-regulation methods. Neurobiological measurements are projected to provide evidence of favorable modifications in autonomic nervous system functioning, indicating beneficial biological responses to anxiety in both intervention groups. In addition, postintervention evaluations will reflect increased participant satisfaction with the techniques, particularly noting subjective differences in comfort and effectiveness. Yoga techniques are anticipated to be perceived as more relaxing, whereas VR may be seen as more stimulating.

In summary, the expected outcomes involve a reduction in anxiety symptoms, enhancement of life quality, and adaptation of emotional regulation strategies, underscoring the efficacy of both intervention methods in addressing performance anxiety among young individuals. This research may significantly contribute to the mental health field by providing relevant data for effective therapeutic strategies and new avenues of study.

## Discussion

### Anticipated Findings

This study aims to test whether 2 types of psychological interventions could significantly improve mental health for young people. First, we expect that implementing VR techniques will significantly reduce anxiety symptoms soon after starting the program, and second, we expect that yoga practices could have long-term significant benefits. These hypotheses align with previous research findings indicating short- and long-term benefits for mental health. Research has shown that both VR and yoga can effectively reduce anxiety symptoms.

For example, VR interventions and particularly those based on CBT have been widely recognized as an effective treatment for anxiety disorders, including performance anxiety. Wu et al [42] indicated that VR-assisted CBT significantly decreases anxiety symptoms compared to traditional methods. Other studies focusing on specific types of anxiety (eg, social anxiety) indicated that VR-CBT effectively reduced anxiety levels in patients with generalized SAD, allowing them to practice social interactions in a safe environment [36]. In addition, exposure-based CBT, which is a core component of VR-CBT, has been validated as the gold standard for treating anxiety disorders, including performance anxiety [43]. This method involves gradual exposure to anxiety-provoking situations, which is facilitated by the virtual environment, making it less intimidating for patients [44]. In conclusion, virtual therapy using cognitive behavioral techniques presents a robust approach for managing performance anxiety. The combination of VR-CBT’s immersive exposure methods and cognitive restructuring strategies offers a comprehensive framework for addressing the cognitive and behavioral aspects of anxiety. As digital health solutions continue to evolve, the integration of VR in therapeutic settings holds significant promise for enhancing the accessibility and effectiveness of mental health interventions.

Yoga-based psychoemotional support intervention provides a holistic approach to managing emotions and stress. According to studies, breathing techniques and yoga poses can contribute to a significant reduction in stress and anxiety levels, thus improving QoL. According to Brown and Ryan [45], mindfulness cultivated through meditation, a central component of yoga, can reduce anxiety and enhance overall life satisfaction. Research by Cramer et al [7] has shown that regular participation in yoga classes is associated with a significant reduction in depressive and anxiety symptoms. This highlights that yoga practice not only improves physical health through poses and breathing techniques but also plays an essential role in enhancing emotional health.

Controlled breathing, known as pranayama, is a central element in yoga practice, with beneficial effects on the autonomic nervous system. According to Jerath et al [46], slow pranayamic breathing techniques induce a physiological response that promotes the activation of the parasympathetic nervous system, associated with states of relaxation and regeneration. Deliberately manipulating the breath generates a hyperpolarization current in the nervous system, facilitating a state of calm and reducing emotional reactivity. Melnychuk et al [47] investigated the connection between breathing and attention, concluding that controlled breathing positively influences cortical functioning and neurophysiological balance. This discovery is essential for understanding the mechanisms by which yoga and pranayama can improve mental well-being and help effectively manage stress.

Yoga practice impacts not only psychological health but also neurological health. Măgurianu and Măgurianu [48] presented the hypothesis that breathing techniques can stimulate processes of angiogenesis and neurogenesis, thus promoting neuronal regeneration. In neuroscience, the law by Hebb states, “neurons that fire together, wire together.” This explains how constant yoga practice, by repeatedly activating the neural groups responsible for well-being states, can facilitate the formation of durable neural networks, thus contributing to brain regeneration and improved cognitive functioning.

By applying the basic principles of Yama and Niyama, combined with techniques of asana (poses), pranayama (controlled breathing), and meditation, yoga offers a practical set of tools for emotion management and stress reduction. Its benefits are not only limited to mental health but also include positive influences on physiological and neurological functioning. These techniques, although accessible to any practitioner, offer significant benefits only in the context of continuous commitment and disciplined practice. Integrating these principles into psychoemotional interventions can yield valuable results in reducing anxiety and improving QoL, demonstrating the holistic potential of yoga as a method of balancing mind and body.

Regarding the effectiveness of yoga practices for anxiety performance, these practices have been associated with significant reductions in anxiety, as they promote relaxation and mindfulness, which are crucial for managing stress [49]. The comparative effectiveness of these interventions suggests that while VR may offer immediate relief, yoga’s benefits may accrue over time, necessitating multiple sessions for optimal results [49]. Both interventions are anticipated to enhance emotional regulation abilities, particularly through the development of cognitive reappraisal strategies. Cognitive reappraisal, a strategy that involves changing one’s interpretation of a situation to alter its emotional impact, has been linked to improved emotional outcomes [50,51]. Studies indicate that individuals who engage in yoga practice report using cognitive reappraisal more frequently, which can lead to better emotional management in stressful situations [52]. Furthermore, the neurobiological mechanisms underlying cognitive reappraisal have been extensively studied, revealing that this strategy activates brain regions associated with emotional regulation, such as the prefrontal cortex [50]. This suggests that participants in both the VR and yoga groups may experience enhanced emotional regulation capabilities after the intervention. In summary, the implementation of both VR techniques and yoga practices is hypothesized to yield significant reductions in anxiety symptoms, enhance emotional regulation abilities, and improve overall QoL. The evidence suggests that while VR may provide immediate relief, yoga’s long-term benefits are substantial, particularly in fostering effective cognitive reappraisal strategies. Future research should continue to explore these interventions’ comparative effectiveness and their underlying mechanisms to optimize therapeutic approaches for anxiety management.

### Future Directions

The results from this study can facilitate the implementation of VR-CBT and yoga intervention to address performance anxiety in students. Through a standardized framework for comparing the 2 interventions, we hope to add value to the expanding field of digital and integrative mental health therapies. As anxiety is increasingly common in young adults, particularly those within academic settings, our research could guide targeted therapeutic approaches that are tailored to a specific person’s needs and preferences.

More generally, this research highlights the need for new, accessible forms of mental health support. VR-CBT is an emerging digital mental health intervention, and it enables individuals to access and explore anxiety in virtual, safe environments. The integration of cognitive restructuring techniques within virtual therapy further enhances its efficacy. Cognitive restructuring helps individuals identify and modify negative thought patterns that contribute to their anxiety. By combining these techniques with VR, patients can visualize and interact with their fears, which has been shown to foster cognitive change and reduce anxiety. For instance, a study highlighted the effectiveness of VR-CBT in helping participants confront and alter their automatic negative thoughts through immersive experiences [53]. Finally, the convenience and accessibility of digital platforms for delivering CBT are crucial in addressing the barriers many individuals face in accessing traditional therapy. Internet-based CBT programs have been shown to significantly reduce psychological distress among patients with anxiety disorders, making them a viable alternative for those unable to attend in-person sessions [54]. This is particularly relevant in the context of the COVID-19 pandemic, where many individuals experienced heightened anxiety levels and barriers to traditional mental health services [55].

In contrast, yoga is a more conventional, all-encompassing discipline that enhances mental health through physiological and psychological processes that may have long-term effects. Finally, our findings could help design individualized mental health programs; VR-CBT might be delivered for people who require instantaneous anxiety relief, and yoga would support long-term emotional stability and health. Future research should address larger, more varied samples or apply these interventions to broader therapeutic contexts to further explore their synergy.

## Appendix

Multimedia Appendix 1 Peer review comments and response from the Academia Romana prin Fundatia Patrimoniu (Romanian Academy Heritage Foundation).

## Abbreviations

AE: adverse event
CBT: cognitive behavioral therapy
DSMB: data safety monitoring board
IRB: institutional review board
PI: principal investigator
QoL: quality of life
RCT: randomized controlled trial
SAD: social anxiety disorder
STAI: State-Trait Anxiety Inventory
VR: virtual reality
VR-CBT: virtual reality–assisted cognitive behavioral therapy

## Data Availability

Details regarding the data safety monitoring board’s charter, reporting procedures, and decision-making framework are outlined in this study’s ethics review documentation and can be made available upon request. Details on data access and sharing policies will be included in the final study report and made available upon request. The deidentified participant-level dataset will be shared upon reasonable requests from qualified researchers, subject to institutional data-sharing policies, ethical considerations, and participant privacy protections.
