# Supplementary material for: Comparative Efficacy of Virtual Reality–Assisted Cognitive Behavioral Therapy Versus Yoga-Based Interventions for Reducing Performance Anxiety in Students: Protocol for a Randomized Controlled Trial
Source: JMIR Res Protoc. 2025 Jun 30;14:e66112. doi: 10.2196/66112 (PMC12260468; doi:10.2196/66112)
Supplement: Multimedia Appendix 1 [file resprot_v14i1e66112_app1.pdf]

Dear Committee,

We thank you for all your recommendations. Below are our responses to each of them, as requested:

1. **Please include a clear statement that participation in the research is voluntary and that participants may withdraw at any time (including their related data) without any consequences for the medical care they are entitled to receive.** We have added these details in the informed consent form, in both versions.

2. **Please specify the duration of the study and the duration of each participant's involvement in the study (e.g., the timeline for the 10 sessions).** We have highlighted in yellow and added track-changes to indicate the session durations and the management of participants who exceed this period in the description of the "Psycho-emotional Assistance Intervention" for both programs.

3. **Please insert the following statement: "There is no commercial interest for any member of the research team in conducting this study."** We have added this detail in the informed consent form, in both versions.

4. **Please add a section that clearly explains the difference between a research initiative and routine medical care. Make any modifications if necessary:**

"You are invited to participate in a research study. The purpose of this study is to generate scientific knowledge that may contribute to improving medical treatments in the future. It is important to understand that participation in this research differs from receiving routine medical care. There are differences between research and routine medical care. Routine medical care aims to provide you with the best possible treatment for your individual health condition. In contrast, the primary goal of this research study is to gather data and information that could benefit future patients and contribute to medical science. Some assessments you may undergo as part of this study (such as questionnaire administration) might not be part of the standard care for your condition. These are specifically designed for research purposes and may include additional evaluations. In this study, your primary care physician is also an investigator, meaning they have dual responsibilities: to care for your health and conduct research. Although they will always prioritize your safety and well-being, their role in this study involves data collection and adherence to research protocols that may not be part of standard medical care. By participating in this research, you are contributing to advancing medical knowledge. Your involvement is entirely voluntary, and you may choose to withdraw at any time without any impact on your standard medical care. Your decision to participate or not will not affect the quality of care you receive from your doctor."

We have added these details in the informed consent form, in both versions, in the section describing participation in sessions for psycho-emotional assistance programs using virtual reality or yoga.

5. **Please clarify what is meant by the expression "diagnosed within the spectrum of anxiety-related mental disorders." Anxiety symptoms can appear in a patient suffering solely from anxiety, as well as in a psychotic patient. We are interested in the inclusion criteria for the study to clarify if there are patients without decision-making capacity.**

We have enhanced the description of the inclusion and exclusion criteria for research participation. For inclusion criteria, we have specified the diagnosis and provided details on various specific cases applicable to the academic setting regarding symptoms and disorders that we can include. For exclusion criteria, we added a clear list of all exclusion criteria and detailed these in an ANNEX to the informed consent form, in both versions, for cases where participants are assigned to the experimental condition of "virtual reality."

6. **Please draft a version of the informed consent (IC) form for the parents of minors included in the study!** We have created a version of the informed consent form for parents or legal guardians, attached as a separate document.

We await the modified version of the IC form. Please make these changes in red or with track changes to expedite the issuance of the Ethics Committee approval.

Thank you very much, and we assure you of our best intentions.

The Research Ethics Committee Team

REVIEWED: Informed Consent within the Project: Applicability of Virtual Reality Techniques vs. Yoga Techniques in the Spectrum of Anxious Valences of Mental Disorders (VRvs.HY)

PARTICIPANT FORM

You are invited to participate in research that tests the extent to which psycho-emotional assistance programs using digital tools (e.g., virtual reality) or programs based on practicing postures and breathing exercises (e.g., yoga) can help manage the specific worries associated with emotional difficulties you may be experiencing (e.g., anxiety). By completing this form and agreeing to participate in the study, you confirm your consent and may also receive information on research reports obtained from the analysis.

Below are some details regarding your participation in the study: ⎱ Completing the form requires approximately 7-10 minutes. ⎱ First, you will be asked to provide some personal information (e.g., age, gender, medical data, educational level). This information is confidential and will only be used to describe participants in this study. ⎱ You will be asked to answer a series of questions related to the anxiety you experience, how you usually manage it, and your experience after participating in one of the psycho-emotional assistance programs following each session. ⎱ You will participate in 10 sessions in one of the available psycho-emotional assistance programs: either virtual reality or yoga. It is important to understand that participating in this research differs from receiving routine medical care or psychological counseling. There are differences between research and medical or psychological care. Routine medical care or psychological counseling aims to provide the best possible treatment for individual health conditions, while the primary goal of this research is to collect data and information that may benefit future patients and contribute to medical and interdisciplinary science. Some measurements (e.g., administering questionnaires) may not be part of standard care; these are specifically designed for research purposes and may include additional evaluations. In this study, medical specialists and psychologists also act as investigators. This dual role means they are responsible for both healthcare and research. While prioritizing the safety and well-being of participants, their role in the study involves data collection and adherence to research protocols. Participation contributes to advancing knowledge in medical and social sciences. Involvement is entirely voluntary, and you may withdraw at any time without any impact on standard care. The decision to participate or not will not affect the quality of care you receive. ⎱ If it is determined during the sessions that your health or psychological condition is affected due to participating in the intervention sessions (e.g., issues from visual stimulation, mobility issues from physical exercises), the physician and psychologist will stop the session and recommend a medical consultation or seek medical assistance for you in case of emergencies. ⎱ This study involves minimal medical and psychological risk. For example, participation in an intervention using virtual reality devices may cause mild discomfort, and those specific to yoga interventions may lead to mild physical discomfort. There are no other known risks associated with these procedures. ⎱ Possible benefits: alleviation of anxiety symptoms associated with a psychiatric disorder and improvement in managing anxious symptoms. ⎱ Responses are used confidentially, without identifying you personally as the form respondent. Since the research involves data collection over multiple stages, your email address is needed for follow-up at 1-2 months. ⎱ Your responses will only be accessible to the principal researchers: CS III Dr. Liviu-Adrian Măgurianu, CS Dr. Cristina Maria Tofan, PhD student Alexandru Marcel Găină, Alexandra Maria Găină, and Magdalena Axinte. ⎱ Study results will be used to adapt student training in line with practical needs, for presentations at

scientific events in psychology and medical fields, and in scientific publications and science dissemination. Responses will be coded for qualitative and quantitative processing following Romania's ethical and legislative rules specific to social research. There are no other intended uses for the results beyond those already presented. The research team has no commercial interest in conducting this research. You have the option to request detailed information about the study results and their significance. For any questions or additional information, feel free to contact us at the email addresses provided: liviumagurianu@yahoo.com. The research is funded through GAR-2023-2025, Romanian Academy Grants, within the research project: Applicability of Virtual Reality Techniques vs. Yoga Techniques in the Spectrum of Anxious Valences of Mental Disorders (VRvs.HY). No financial or other form of reward is offered for participation. Travel, accommodation, and other expenses are not covered. There are no financial or other costs for participating in this study. Any new information that may arise during the study that could affect your willingness to continue participating will be communicated to you immediately.

Since some data will be collected online, here are some details for data collection through online forms such as Google Forms. **PERSONAL DATA PROCESSING:** In compliance with the requirements of Regulation (EU) 2016/679 on the protection of individuals regarding the processing of personal data and the free movement of such data and repealing Directive 95/46/EC (General Data Protection Regulation) and Law no. 506/2004 on the processing of personal data and privacy protection, the research team assumes the obligation to manage the data you provide in safe conditions and only for the specified purposes, whether it concerns email address, socio-demographic data, or questionnaire responses. Data will be collected and stored online on the Google Drive platform, which complies with the requirements of Regulation (EU) 2016/679 on the protection of individuals regarding personal data processing and the free movement of such data, as well as repealing Directive 95/46/EC (General Data Protection Regulation).

I have read and understood this consent form and voluntarily agree to participate in the described study. Participation is voluntary, and you may withdraw at any time without consequences for the medical and psycho-emotional support you are entitled to. Upon withdrawal, the data collected up to that point will be deleted. I receive a copy of this form.

Date:

Participant's Agreement: ....

Informed Consent within the Project:

Applicability of Virtual Reality Techniques vs. Yoga Techniques in the Spectrum of Anxious Valences of Mental Disorders (VRvs.HY)

PARENT OR LEGAL GUARDIAN FORM

Invitation to Participate: Your child is invited to participate in a study that tests the extent to which psycho-emotional support programs using digital tools (e.g., virtual reality) or programs based on posture and breathing exercises (e.g., yoga) may assist in managing specific worries associated with emotional challenges they face (e.g., anxiety). The purpose of this study is to generate scientific knowledge that may help improve medical treatments in the future. By signing this form, you consent to your child's participation in this study and confirm that you understand the purpose and details of their involvement.

Details of Participation:

- **Duration to complete the form:** 7-10 minutes.
- **Collected Personal Information:** Age, gender, medical data, educational level. This information is confidential and will be used only for participant description purposes.
- **Questionnaires:** Your child will answer questions related to their experience of anxiety, anxiety management, and their experiences following participation in psycho-emotional support programs.
- **Sessions:** Your child will attend 10 sessions in one of the available programs: virtual reality or yoga. It's important to understand that participating in this research differs from receiving routine medical care or psychological counseling. Medical or psychological care aims to provide the best possible treatment for individual health. However, the primary purpose of this research is to collect data that may benefit future patients and contribute to medical and interdisciplinary science. Some assessments (e.g., administering questionnaires) may not be part of standard care, as these are specifically designed for research purposes and may include additional evaluations. In this study, medical specialists and psychologists also serve as researchers, meaning they have a dual responsibility to care for health and conduct research. Although they always prioritize participants' safety and well-being, their role in the study involves data collection and adherence to research protocols. Participation contributes to the advancement of medical and social sciences knowledge. Involvement is entirely voluntary, and you may withdraw at any time without any impact on standard care. The decision to participate does not affect the quality of care provided.
- **Medical Interventions:** If it is determined that your child's health or psychological condition is affected (e.g., issues from visual stimulation or mobility problems from physical exercises), the session will be stopped, and a medical consultation or emergency medical assistance will be recommended.
- **Risks:** Participation involves minimal risk, such as mild discomfort from virtual reality devices or physical discomfort from yoga exercises.

- **Benefits:** Potential alleviation of anxiety symptoms and improvement in managing these symptoms.
- **Confidentiality:** Responses are confidential. An email address will be required for data collection at various stages. Responses are used confidentially, and your child cannot be personally identified as a respondent. Since research involves data collection at multiple stages, an email address is needed for follow-up at 1-2 months.
- **Data Access:** Only the primary researchers (CS III Dr. Liviu-Adrian Măgurianu, CS Dr. Cristina Maria Tofan, PhD student Alexandru Marcel Găină, Alexandra Maria Găină, Magdalena Axinte) will have access to responses.
- **Use of Results:** Study results will be used to adapt student training according to practical needs, for presentations at scientific events in psychology and medicine, and in scientific publications and science dissemination. Responses to existing questions will be coded for qualitative and quantitative analysis, following Romanian ethical and legal norms specific to social research.
- **Costs and Rewards:** There are no financial or other rewards for participation, nor are there reimbursements for travel or accommodation expenses. Participation does not incur any financial costs.
- **Ongoing Information:** Any new information that may impact participation will be immediately communicated.
- **No Other Purposes:** Results will not be used for purposes other than those already presented.
- **No Commercial Interests:** The research team has no commercial interests in conducting this research.
- **Requesting Information:** You may request detailed information about study results and their significance. For questions or additional information, please contact us at the following email addresses: liviumagurianu@yahoo.com. The research is funded by GAR-2023-2025, Romanian Academy Grants, through the research project: Applicability of Virtual Reality Techniques vs. Yoga Techniques in the Spectrum of Anxious Valences of Mental Disorders (VRvs.HY).

Since some data will be collected online, here are some details regarding data collection through online forms like Google Forms.

PROCESSING OF PERSONAL DATA: In compliance with Regulation (EU) 2016/679 on the protection of individuals regarding personal data processing and the free movement of such data, repealing Directive 95/46/EC (General Data Protection Regulation), and Law No. 506/2004 on processing personal data and privacy, the research team commits to managing the data you provide safely and solely for the specified purposes, whether it concerns email address, socio-demographic data, or questionnaire responses. Data will be collected and stored online on the Google Drive platform, which complies with the requirements of Regulation (EU) 2016/679.

I have read and understood this consent form, and I agree for my child to participate in the described study. Participation is voluntary, and my child may withdraw at any time without consequences for the medical and psycho-emotional care to which they are entitled. In the event of withdrawal, data collected up to that point will be deleted. I receive a copy of this form.

Date:

Parent/Legal Guardian Signature: ....

Appendix: Information Regarding Participation in the Virtual Reality Experimental Group

We strongly recommend using the application only under the supervision of a mental health professional and emphasize the need to strictly follow the instructions provided in the tutorials. Before using the application, please thoroughly read and confirm that you do not suffer from any of the following conditions:

- **A) Medical Conditions:** You must inform the investigator of any medical conditions.
- **B) Neurological Disorders:** Epilepsy and other conditions where visual stimuli trigger seizures, including flashing light-induced photosensitivity or epileptic seizures. Individuals who experience severe migraines, which may also be exacerbated by VR, are contraindicated.
- **C) Visual Impairments:** Individuals unable to see the VR environment clearly within the headset, even with contact lens adjustments or advanced glaucoma.
- **D) Vestibular Disorders:** Excludes Meniere's disease or any other diagnosed vestibular disorder, such as neuritis, if vertigo is easily triggered. If symptoms similar to motion sickness occur, exposure should stop immediately.
- **E) Photosensitivity:** Documented history of photosensitivity or the onset of symptoms like dizziness or nausea following intermittent light exposure.
- **F) Cardiovascular Disorders:** Cardiovascular diseases, including recent or distant myocardial infarction, unstable angina, or uncontrolled high blood pressure, are absolute contraindications as these may be exacerbated by VR exposure.
- **G) Pregnancy:** Excludes individuals in any trimester due to unknown risks of VR-induced stress on fetal development.
- **H) Bio-Psycho-Social Aspects:** Severe mental health disorders contraindicate VR use if dissociation is expected, such as in dissociative identity disorder or acute anxiety disorders with known paroxysmal episodes. Individuals with panic disorders may experience panic attacks due to VR scenarios. Individuals with trauma or PTSD may be at risk from scenarios replicating traumatic encounters. Finally, individuals with suicidal ideation or severe psychomotor retardation may be at risk in VR environments. It is essential to have a safe, controlled environment free of hazards, necessary for VR headset or controller safety.

- **Cognitive and Behavioral Factors:** Cognitive impairments, such as severe intellectual disabilities or dementia, make it difficult to understand the tasks or follow safety instructions. Absence of legal guardianship or consent for minors or adults under guardianship who cannot provide consent and lack a legally involved guardian for supervision.
- **Lack of Adherence:** Individuals with a history of non-compliance with medical or psychological treatment may not adhere to the application tutorials.
- **I) Physical Factors:** Individuals with physical disabilities that prevent the use of standard VR controllers or headsets when unsupervised or without assistance.
- **J) Language Barriers:** Individuals who do not speak or understand the primary language of the VR device and for whom translation services are unavailable. This study currently offers language assistance for Romanian and English-speaking participants.
- **K) Known allergies to any materials used in the headsets or controllers.**
- **Final Note:** If any of the above issues arise during exposure, such as bio-psycho-social aspects or allergic reactions, usage will be stopped, and your primary physician or the lead investigator responsible on the platform at marcel-alexandru_t_gaina@d.umfiasi.ro will be contacted.

I have read and understood this consent form, and I agree for my child to participate in the described study. Participation is voluntary, and my child may withdraw at any time without consequences for the medical and psycho-emotional care to which they are entitled. In the event of withdrawal, data collected up to that point will be deleted. I receive a copy of this form.

Date:

Parent/Legal Guardian Signature: ....
